# Oncolytic Vaccinia Virus in Lung Cancer Vaccines

**DOI:** 10.3390/vaccines10020240

**Published:** 2022-02-04

**Authors:** Cao-Sang Truong, So Young Yoo

**Affiliations:** BIO-IT Foundry Technology Institute, Pusan National University, Busan 46241, Korea; truongcaosang@gmail.com

**Keywords:** vaccinia virus, oncolytic virus, non-small cell lung cancer, cancer vaccine, immunotherapy, personalized vaccination

## Abstract

Therapeutic cancer vaccines represent a promising therapeutic modality via the induction of long-term immune response and reduction in adverse effects by specifically targeting tumor-associated antigens. Oncolytic virus, especially vaccinia virus (VV) is a promising cancer treatment option for effective cancer immunotherapy and thus can also be utilized in cancer vaccines. Non-small cell lung cancer (NSCLC) is likely to respond to immunotherapy, such as immune checkpoint inhibitors or cancer vaccines, since it has a high tumor mutational burden. In this review, we will summarize recent applications of VV in lung cancer treatment and discuss the potential and direction of VV-based therapeutic vaccines.

## 1. Introduction

Lung cancer is the second most commonly diagnosed cancer, with 2.2 million new cases (11.4%), and is the leading cause of cancer death, with an estimated 1.8 million deaths (18%), according to the GLOBOCAN 2020 [1]. It can be categorized into two major types—non-small cell lung cancer (NSCLC) and small cell lung cancer (SCLC). NSCLC accounts for 85% of all cases, while the figure for SCLC is approximately 15%. NSCLC is further divided into lung adenocarcinomas (40%), squamous cell carcinoma (25–30%), and large cell carcinoma (10–15%), based on their histological features [2,3,4]. Early stages of lung cancer usually do not show any symptoms. Most patients with lung cancer are diagnosed at advanced stages (stage III/IV). The average age of diagnosed lung cancer patients is 70 years, with a high incidence of cases from 65 to 75 years of age. Among these patients, 50–70% of patients are diagnosed with locally advanced or metastatic cancer. The International Association for the Study of Lung Cancer (IASLC) reported that five-year survival in NSCLC for stages IIIA, IIIB, IIIC, and IV as 36%, 26%, 13%, and 6%, respectively. This is a challenge for the treatment of lung cancer as the elderly population is not eligible for aggressive therapies due to the age-related functional decline of many organs [5,6]. It has been reported that patients with late stages of lung cancer have shorter life expectancy than those diagnosed at early stages. Therefore, it is critical to develop methods for screening and detection of disease at early stages and new cancer therapies for the treatment of tumor at advanced stages. The treatment of lung cancer depends on the tumor stage at diagnosis. For NSCLC, surgical resection is the standard treatment option for patients at stage I and II. After complete resection, platinum-based adjuvant chemotherapy is recommended for stage II tumors. Patients who cannot undergo resection are offered radiotherapy with curative intent. Patients at advanced stages are offered radiochemotherapy (stage IIIB) and palliative therapies (stage IV) [4,7,8].

Targeted therapy has emerged as an important treatment option for patients with NSCLC. It attempts to interfere with a number of identified major pathways, such as epidermal growth factor receptor (EGFR), PI3K/AKT/mTOR, RAS–MAPK, and NTRK/ROS1 pathways. A number of drugs targeting these pathways have been developed and have shown clinical benefits, including prolonged survival and enhanced quality of life in these patients. Despite the potential effects on specifically targeting tumors, the tumors inevitably develop drug resistance during the treatment [7,8].

The needs for novel therapies to improve survival in lung cancer requires the discovery and development of new treatment modalities for NSCLC patients at advanced stages. Over the last decade, a rapidly growing number of studies in the cancer field have provided a better understanding of the immune system and cancer cells, leading to a new era of cancer immunotherapy, which has revolutionized cancer treatment in NSCLC patients [4,9]. Unlike other cancer therapies, which directly target the tumors, immunotherapy harnesses the immune system of tumor-bearing hosts to combat cancer. Studies on immunotherapy in NSCLC have focused on two immunotherapeutic strategies—immune checkpoint inhibitors (ICIs) and cancer vaccines. The emergence of ICIs has provided an effective treatment option for patients with advanced and metastatic NSCLC. It has shown remarkable benefits and dramatically improved clinical outcomes; however, this has only been shown in a small subset of NSCLC patients. Despite the advances in treatment of lung cancer by ICIs, the majority of patients with metastatic NSCLC are refractory to ICIs in the first line treatment. The response rate of patients is even lower (<20%) in the second line or higher line therapy. This challenge indicates that the immune response has not been fully harnessed yet to provide clinical benefits for a greater number of patients [10,11].

Another immunotherapeutic strategy with great potential to address these obstacles is cancer vaccination, which aims to generate potent immune responses against the immune evasion of tumors. The research in cancer vaccines has been rapidly increasing and has delivered promising results. The safety and efficacy of cancer vaccines in NSCLC has been validated in numerous phase I and phase II clinical trials. However, no positive data has been reported in the late phase clinical trials over the last decade [12,13,14]. The failure of therapeutic cancer vaccines in NSCLC is attributed to the suboptimal vaccine design including inappropriate antigen targeting, inadequate patient selection (stage of disease) [15], ineffective adjuvants [16], tumor-induced immunosuppression, or immunosenescence [17]. These challenges drive a critical need for novel research in tumor immunology and vaccine development to design effective vaccine regimens for treatment of NSCLC.

Recently, oncolytic viruses (OVs) have been used to treat a variety of cancer types, especially at advanced stages. As OVs are able to selectively replicate in and kill tumor cells and subsequently induce systemic anti-tumor immune responses, they can be combined with other immunotherapies, such as ICIs or cancer vaccines, which act on different mechanisms to improve therapeutic anti-cancer effects. Oncolytic vaccinia virus (VV) is currently being used as a novel therapeutic strategy in cancer treatment. The effectiveness of VV-based therapies has been demonstrated in a wide range of cancer types in many studies. Moreover, the research using VV as a potent immune adjuvant for therapeutic cancer vaccines in NSCLC has been focused on inducing robust anti-tumor immune responses against tumors [15,18,19]. Therefore, in this review, we will focus on the recent applications of VV on the treatment of NSCLC and discuss future opportunities of this novel strategy.

## 2. Cancer Vaccines in NSCLC

### 2.1. Mechanism of Action of Cancer Vaccine

**Figure 1:**
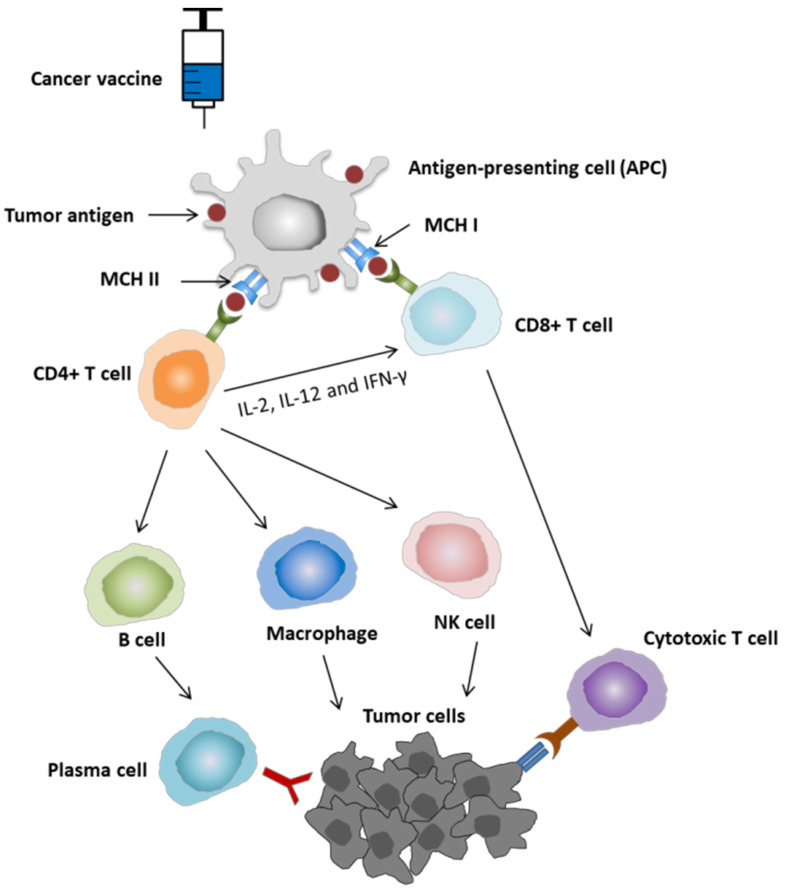
Mechanism of action of therapeutic cancer vaccine. Tumor antigens from vaccines are taken up and processed by antigen-presenting cells (APCs) and presented on MHC class I and MHC class II molecules on the surface of these cells. In the tumor-draining lymph nodes, the interaction of specific T cell receptor (TCR)—the MCH-I complex—lead to the activation of CD8+ T cells and cytotoxic T lymphocytes (CTLs) and subsequently antigen recognition in the tumor cells by CTLs. CTLs then destroy the tumor cells by different processes, such as the secretion of pro-inflammatory mediators (IFNγ, TNFα) or via the perforin/granzyme pathway. The presentation of tumor antigens on MHC-II complex also leads to CD4+ T helper cell activation, which facilitates the activation of several T cell subtypes. The secretion of IL-2, IL-12, and IFN-γ by CD4+ T helper cells promotes the activation of CD8+ T cells into CTLs. In addition, activated CD4+ T helper cells promote tumor clearance by enhancing the generation of antibodies against tumor antigens by B cells, killing the activity of natural killer cells and the phagocytosis of tumor cells by macrophages Abbreviations: MHC—major histocompatibility complex; APC, IL—interleukin; IFN—interferon; NK cell—natural killer cell (re-drawn from the presentation by L. Decoster et al. [20]).

Cancer vaccines can be used for both the prevention and treatment of cancer [14]. Cancer vaccines may induce cellular and humoral immune responses against tumor-specific or associated antigens. The injection of a cancer vaccine results in a cellular immune response, which begins with the uptake of the tumor-associated antigens (TAAs) by antigen-presenting cells (APCs), such as dendritic cells (DCs). The TAAs are then processed and displayed in the surface of these cells in association with major histocompatibility complex (MHC) class I and class II. The interaction of tumor antigens on MHC complexes with T cell receptors (TCRs), specific to T cells, leads to the activation of CD4+ T helper cells and CD8+ T cells. CD4+ T helper cells also enhance the immune response by secreting IL-2, IL-12, and IFN-γ, leading to the activation of CD8+ T cells and cytotoxic T lymphocytes (CTLs). The CTLs recognize the antigens on tumor cells and subsequently induce apoptosis of tumor cells (cellular immune attack). In addition, activated CD4+ T cells enhance the killing activity of natural killer cells the phagocytic activity of macrophages, and stimulate B cells, leading to the production of antigen-specific antibodies by differentiated plasma cells (humoral immune attack). These antibodies can bind to and neutralize tumor antigens, mediating the tumor clearance (Figure 1) [20,21,22].

### 2.2. Why Are Cancer Vaccines Needed in the Treatment of NSCLC?

Surgery, chemotherapy, and radiotherapy are well-recognized cancer treatments for NSCLC at early stages. However, these approaches are ineffective in advanced or recurrent stages. Harnessing the immune system is the most powerful therapeutic strategy to fight NSCLC due to its high tumor mutational burden (TMB) [8]. Particularly, high TMB is associated with increased expression of tumor-specific antigens (neoantigens), which can be recognized by the immune system; therefore, high TMB increases the probability of recognition and elimination of tumor cells by the immune system, and thus improves the response to cancer immunotherapy [23,24,25]. The applications of immunotherapy have brought new breakthroughs in the treatment of NSCLC, and have improved clinical benefits of patients with NSCLC. Currently, ICIs are the only approved immunotherapy option for NSCLC [8]. However, the current treatment for NSCLC with ICIs is suboptimal, as the efficacy of the therapy is limited to a small subset of patients [10,12,13]. The reasons for the low response rate of NSCLC patients remain poorly understood; therefore, there is a high clinical need for exploring a new treatment modality.

Therapeutic cancer vaccines offer a novel promising immunotherapeutic intervention for the treatment of patients with NSCLC [26,27]. Therapeutic cancer vaccines are designed to instruct the immune system to specifically target tumor antigens and selectively fight against cancer cells [28]. Subsequently, the cancer vaccines are able to create long-lasting immunological memory to control tumor growth and prevent recurrence, minimizing non-specific or adverse events. Therefore, cancer vaccines may provide a safer and more effective therapeutic option than other therapies in cancer treatment [11,29]. A number of cancer vaccines are designed to overcome the existing challenges of NSCLC treatment and are currently undergoing in phase III clinical trials. The growing number of studies in this field may increase the likelihood of success of cancer vaccines in improving the clinical outcome of NSCLC patients.

### 2.3. Current Cancer Vaccines for NSCLC

Many clinical studies have been conducted to examine the efficacy and safety profile of various cancer vaccines in advanced-stage NSCLC, and have achieved promising results in early phases of clinical trials. The therapeutic cancer vaccines for NSCLC includes allogeneic whole-cell vaccines, peptide or protein vaccines, DNA vaccines, and vector-based vaccines (Table 1).

Allogeneic whole-cell vaccines are vaccines that use cancer cells isolated from a patient, which are modified and processed, and then administered to another patient to induce cytotoxic immune response to a similar cell type.

Belagenpumatucel-L is an allogenic whole-cell vaccine that is comprised of 4 irradiated NSCLC cell lines (H460, RH2, SKLU-1, H520) transfected with TGF-ß2 antisense plasmid. The efficacy and safety of this vaccine were demonstrated in two phase II clinical trials in advanced NSCLC patients (stage IIIA/IIIB or IV) in 2006 and 2009. However, the phase III clinical trial failed to show improved overall survival [14,30].

Another example of the allogeneic vaccine is autologous or allogeneic NSCLC cells plus GM.CD40L-expressing K562 cells. The phase I clinical trials of irradiated autologous tumor cells plus GM.CD40L bystander cells was conducted in stage IV cancer patients, including NSCLC and SCLC patients. Although there was no tumor regression observed after vaccination, this study demonstrated that the GM.CD40L bystander-based vaccine can activate tumor-specific T cell responses without exhibiting any toxicity [31]. The phase II clinical trial was designed to evaluate the efficacy, safety, and immunogenicity of the vaccine that combined irradiated allogeneic lung adenocarcinoma cells with a bystander K562 cell line, transfected with hCD40L and hGM-CSF. The trial did not meet the primary endpoint of inducing radiologic tumor regression, even in patients with observed immune response [32]. To test the effectiveness of GM.CD40L vaccine in combination with an adjuvant CCL21 in patients with advanced lung adenocarcinoma, a phase I/II randomized trial was carried out; however, improved overall survival and progression-free survival were not observed [33].

Peptide or protein vaccines are made from TAA proteins or short fragments of TAA proteins called TAA-derived peptides. These peptides contain epitopes that can be presented by MHC molecules at the cell surface and recognized by T cells.

CIMAvax epidermal growth factor (CIMAvax-EGF) vaccine is one of the most well-known protein vaccines for NSCLC treatment. The vaccine was developed in Cuba and its use was approved in Cuba, Venezuela, and Peru for the treatment of stage IIIB and IV NSCLC, progressed after a first line of chemotherapy [14]. CIMAvax-EGF vaccine consists of a chemical conjugate of the EGF with an adjuvant, the P64 protein derived from the Meningitis B bacteria and Montanide ISA 51. In a phase II clinical trial, the CIMAvax-EGF vaccine was demonstrated to be safe and immunogenic in NSCLC patients at stage IIIB/IV of the disease after the complete first-line chemotherapy [34]. More importantly, a phase III clinical trial was conducted in NSCLC patients at stage IIIB/IV NSCLC after completing first-line chemotherapy to evaluate overall survival, safety, immunogenicity, and EGF concentration in serum after the CIMAvax-EGF vaccine. The data showed a significant improvement in survival benefit in the group that received four doses of the vaccine. The CIMAvax-EGF vaccine was not only immunogenic but it also reduced the EGF concentration to undetectable levels [35]. A phase IV study was conducted in patients with NSCLC after a complete front-line chemotherapy or in patients who were unfit for chemotherapy in 65 Policlinic areas and 16 hospitals in Cuba over 3 years. Subsequently, no significant differences in median overall survival were observed between vaccinated patients and short- and long-term survivors [36].

MAGE-A3 is the first discovered cancer/testis antigen (CTA) that is considered as a neoantigen when expressed in cancer cells, and possesses the ability to provoke cancer-specific immune responses. MAGE expression is observed in 30–50% of NSCLC tissue samples; therefore, it has high specificity for recognition as tumor marker [37]. The efficacy of MAGE-A3 as adjuvant therapy was examined in a randomized, double-blind, placebo-controlled MAGRIT phase III trial, showing that adjuvant therapy with the MAGE-A3 did not show the benefit in disease-free survival compared with placebo in patients with resected MAGE-A3-positive NSCLC. Therefore, the development of MAGE-A3 for the use of NSCLC treatment has been stopped [38].

NY-ESO-1 is another well-studied CTA and is considered as a highly immunogenic antigen to induce integrated humoral and cellular responses [39]. NY-ESO-1 is expressed in 25–30% in NSCLC samples [40,41]. In a study to evaluate the association of NY-ESO-1 with chemotherapy response, NY-ESO-1 was suggested as a predictive factor for increased response to neoadjuvant chemotherapy and benefit from adjuvant chemotherapy [40]. Although several trials studying NY-ESO-1 vaccines with different adjuvants showed positive results with the activation of immune response, NY-ESO-1-based vaccine development has faced a dilemma since the clinical outcomes were not improved [42].

Racotumomab (formerly known as IE10) is a therapeutic vaccine based on a monoclonal anti-idiotypic antibody developed in Cuba for NSCLC treatment. Racotumomab was also registered in Cuba and Argentina with the trade name Vaxira for the treatment of patients with advanced-stage NSCLC. Racotumomab stimulates cell death by inducing a specific humoral and cellular immune response against the NeuGcGM3 ganglioside present in tumor cells [5]. In 2014, a randomized, double-blind, placebo-controlled phase II/III clinical trial was conducted to evaluate the efficacy of racotumomab vaccine in switch maintenance in NSCLC patients at stage IIIB/IV. The results showed that the clinical outcomes, such as overall survival and progression-free survival, were improved in the vaccinated patients, with mild or moderate adverse effects [43]. In 2021, to assess the use of the racotumomab vaccine as switch maintenance and second-line therapy for patients with NSCLC in routine clinical practice, a study was carried out in advanced-stage NSCLC, indicating that racotumomab in routine clinical practice prolonged overall survival in NSCLC patients. Racotumomab is, therefore, an option for switch maintenance for patients with NSCLC [5].

BLP25 liposome vaccine (L-BLP25) is a 20 amino acid peptide. It is designed to target MUC1, which is overexpressed and aberrantly glycosylated in NSCLC, and induce a cellular immune response that may lead to immune rejection of tumor tissues that express the MUC1 antigen. The effects of L-BLP25 on survival and toxicity in patients with stage IIIB and IV NSCLC were investigated in a randomized phase IIB trial, indicating that the difference in survival between patients who received L-BLP25 plus best supportive care (BSC) or BSC alone was not statistically significant; although, the median survival time was 4.4 months longer in the patients assigned to the L-BLP25 arm with no significant toxicity [44]. In 2014, a phase III START trial was conducted to assess the effectiveness of tecemotide (L-BLP25) in improved survival in patients with stage III unresectable NSCLC after chemoradiation. However, the study showed no significant difference in overall survival in the group which received tecemotide after chemoradiotherapy compared with the placebo group [45].

DNA vaccines contain DNA that codes for specific antigens to elicit adaptive immunity. Elenagen is a plasmid encoding p62/SQSTM1. The efficacy and safety of Elenagen was evaluated in a first-in-human, multicenter I/IIa trial in patients with advanced solid tumors, including lung cancer. The results showed that Elenagen exerted anti-tumor activity in a range of solid tumors. In particular, 12/27 patients, including 1 lung cancer patient, achieved stable disease for at least 8 weeks with a good safety profile [46].

TG4010 (MVA-MUC1-IL-2) is a vector-based vaccine that uses Ankara virus, an attenuated, genetically modified vaccinia virus, to express MUC-1 and IL-2. The vaccine was developed for cancer patients whose tumors express the MUC1 antigen. A phase I clinical trial was conducted to examine the safety profile and appropriate dose of the vaccine for further trials. A total of 4 out of 13 patients with different solid tumors achieved stable disease for 6–9 months, and 1 lung cancer patient showed a significant decrease in the metastatic tumor size that lasted for 14 months [47]. In a phase II study conducted in patients with stage IIIB/IV NSCLC, the combination of TG4010 with first line chemotherapy was assessed, showing encouraging results in the median time to progression and median overall survival [48]. In another phase IIB clinical trial, evaluating the combination of TG4010 with first-line chemotherapy in advanced NSCLC, TG4010 was demonstrated to enhance the effect of chemotherapy, which was shown in the improved 6-month progression-free survival (PFS) [49]. In addition, the improved PSF of this combination was confirmed in a phase IIB part of a randomized, double-blind, placebo-controlled, phase IIb/III trial [50].

**Table 1:** Current cancer vaccines for lung cancer.

Type of Vaccine	Vaccine	Tumor Stage	Phase Trial	Patients	Time	Results	Adverse Events (AEs)	Reference
Allogeneic vaccines	Belagenpumatucel-L	NSCLC II, IIIA, IIIB and IV	II	75	2006	Belagenpumatucel-L is well tolerated, and the survival advantage justifies further phase III evaluation.	Non-significant.	[12]
		NSCLC IV	II	21	2009	Overall survival was 562 days.	Non-significant.	[13]
		NSCLC III/IV	III	532	2015	No difference in survival between the arms. No differences in progression-free survival.	No serious AEs.	[30]
	Autologous or allogeneic NSCLC cells plus GM.CD40L-expressing K562 cells	NSCLC IV	I	21	2007	There was no tumor regression after vaccination, but many patients had stable disease.	No toxicity.	[31]
		Refractory advanced stage	II	24	2013	The primary endpoint, inducing radiologic tumor regression, was not reached. Median OS was 7.9 months and median PFS was only 1.7 months.	Common toxicities were headache and site reaction.	[32]
Peptide or protein vaccines	CIMAvax-EGF	IIIB/IV	II	80	2008	Good anti-EGF antibody response was obtained in 51.3% of vaccinated patients.	Less than 25% of cases and were grade 1 or 2	[34]
		IIIB/IV	III	405	2016	Survival benefit was significant: Median survival time (MST) was 12.43 months for the vaccine arm versus 9.43 months for the control arm. MST was higher (14.66 months) for vaccinated patients with high EGF concentration at baseline.	Long-term vaccination is safe. Most frequent adverse reactions were grade 1 or 2 injection-site pain, fever, vomiting, and headache.	[35]
	MAGE-A3	IB, II, and IIIA MAGE-A3-positive NSCLC	III (MAGRIT)	13.849	2016	Adjuvant treatment with the MAGE-A3 immunotherapeutic did not increase disease-free survival. Further development of the MAGE-A3 immunotherapeutic for use in NSCLC has been stopped.	The most frequently reported grade 3 or higher adverse events were infections and infestations, vascular disorders, and neoplasm.	[38]
	Racotumomab (IE10)	NSCLC IIIB/IV	II/III	176	2014	Median progression-free survival (PFS) in vaccinated patients was 5.33 versus 3.90 months for placebo.	The most common adverse events in the racotumomab-alum arm were burning and pain at the injection site, bone pain, and asthenia.	[43]
	BLP25 liposome vaccine	NSCLC IIIB/IV	IIB	171	2005	The survival difference of 4.4 months observed with the vaccine did not reach statistical significance.	Non-significant.	[44]
		NSCLC III	III (START)	1239	2014	No significant difference in overall survival.	Serious adverse events with a greater than 2% frequency with tecemotide were dyspnea, metastases to central nervous system and pneumonia.	[45]
DNA vaccines	Elenagen	Advanced solid tumors	I/IIA	27	2017	Most of the patients achieved stable disease for at least 8 weeks.	No severe AEs.	[46]
Vector-based vaccines	TG4010	Different solid tumors	I	13	2003	A total of 4 of the 13 patients achieved stable disease. One lung cancer patient who was initially progressing after the first injections later showed a marked decrease in the size of his metastases that lasted for 14 months.	Injection site pain and influenza-like symptoms.	[47]
		NSCLC III/IV	II	65	2008	The median overall survival was 12.7 months for arm 1 (combined TG4010 with chemotherapy) and 14.9 for arm 2 (vaccine alone).	Mild–moderate injection site reactions, flu-like symptoms, and fatigue being the most frequent adverse reactions.	[48]
		NSCLC IIIB/IV	IIB	148	2011	6-month progression-free survival (PFS) was 43.2% in the TG4010 plus chemotherapy group, and 35.1% in the chemotherapy alone group	Common AEs include fever, abdominal pain, and injection-site pain. The most common grade 3–4 AEs were neutropenia, and fatigue. Anorexia and pleural effusion were grade 3–4 AEs that differed significantly between groups.	[49]
		NSCLC IV	IIB/III	222	2016	The combination of TG4010 with chemotherapy seems to improve PFS relative to placebo plus chemotherapy.	No grade 3–4 or serious AEs deemed to be related to TG4010 only; 4 (4%) patients presented grade 3 or 4 AEs related to TG4010 and other study treatments. The most frequent severe AEs were neutropenia, anemia, and fatigue.	[50]

## 3. Recent Applications of Vaccinia Virus for NSCLC Treatment

### 3.1. Vaccinia Virus

**Figure 2:**
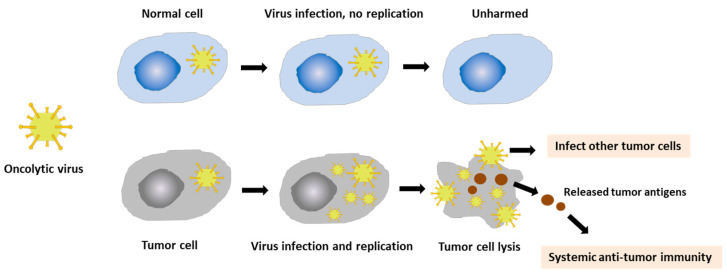
Mechanism of action of oncolytic virus. After the oncolytic viruses (OVs) enter normal cells, the cells stimulate different signaling pathways to limit virus spread and promote rapid cell death and the viral clearance. The virus is not able to replicate in the normal cells, leaving them unharmed. In cancer cells, OVs replicate in and lyse the cancer cells, which can directly destroy tumor cells. In addition, the release of tumor antigens and other danger signals following cell death initiates a systemic anti-tumor immune response that promotes tumor regression at distant tumor sites that are not exposed to OVs.

Oncolytic virotherapy (OVT) is a form of immunotherapy in which oncolytic viruses (OVs) are used to kill the cancer cells, while leaving normal cells unharmed. OVs selectively replicate in and lyse the cancer cells, inducing a systemic anti-tumor immune response (Figure 2). OVT has shown promising results in cancer treatment over the last two decades. OVs have been widely used for cancer treatment, especially advanced-stage cancers, which do not respond to conventional therapies, such as chemotherapy or radiotherapy. OVs are considered as powerful therapeutic agents as they provide good efficacy, fewer side effects, and are less harmful to cancer patients. Currently, several viruses, including vaccinia virus, coxsackievirus, adenovirus, reovirus, herpes simplex virus, measles virus, and maraba virus, are being investigated for use in the treatment of different types of advanced cancers [18,19,51]. 

In normal cells, following the entry of the virus into the cells, numerous signaling pathways are activated to detect and clear pathogenic viral particles. The antiviral machinery can be triggered to limit viral spread and target infected cells for apoptosis or necrosis. Therefore, the OVs cannot replicate in normal cells. However, the process is disrupted in cancer cells, supporting viral replication [52]. Many OVs enter the cancer cells via receptors presenting in the host cells; however, the entry of vaccinia virus occurs via different mechanisms [53]. VVs primarily enter into the cells by endocytosis.

Vaccinia virus (VV) is a member of the orthopoxvirus genus of the Chordopoxvirinae subfamily. VV has a linear, double-stranded DNA genome approximately 192 kb in length, which encodes about 200 genes. The entire VV life cycle occurs within the cytoplasm of mammalian cells. Three forms of virus exist during the life cycle of VV, including intracellular mature virion (IMV), cell-associated enveloped virion (CEV), and extracellular enveloped virion (EEV). IMV and EEV are seen most often during assembly. While IMV enters the cells by fusion with the plasma membrane, EEV enters the cells by endocytosis [53,54]. There are three major strains of VV that have been characterized to date, including Lister, Western Reserve, and Wyeth. VV has several advantages that make it a promising agent for OVT. VV has played a critical role in the success of the vaccine against smallpox, one of the deadliest diseases in human history for over a century. The long and extensive history of the use of VV in humans has suggested that it is a safe oncolytic agent. Furthermore, the large genome enables the insertion of a huge amount of foreign DNA without significantly reducing the replication ability of the virus. A different feature of VV from other classes of DNA viruses is that VV remains in the cell cytoplasm for the duration of the infectious cycle.

As VV mainly relies on its own encoded proteins for processes involved in DNA replication and mRNA synthesis, it can replicate in numerous cell types and avoid host defense mechanisms due to limited interaction with host proteins. Compared with other OVs that are limited to a number of animal models, VV has a broad host range [53,55,56,57,58]. This advantage facilitates the use of VV to study in laboratory animal models, and easily translates into clinical trials. In addition, the natural tropism to cancer cells is also considered as a favorable feature of VV compared with other oncolytic viruses. VV has been used as platforms of many exploratory approaches to treat cancer. These approaches include the following: (1) a delivery vehicle for anti-cancer transgenes; (2) a vaccine carrier for tumor-associated antigens and immunoregulatory molecules in cancer immunotherapy; (3) an oncolytic agent that selectively replicates in and lyses cancer cells [54,59,60,61,62].

### 3.2. Vaccinia Virus for the Modulation of Tumor Microenvironment

**Figure 3:**
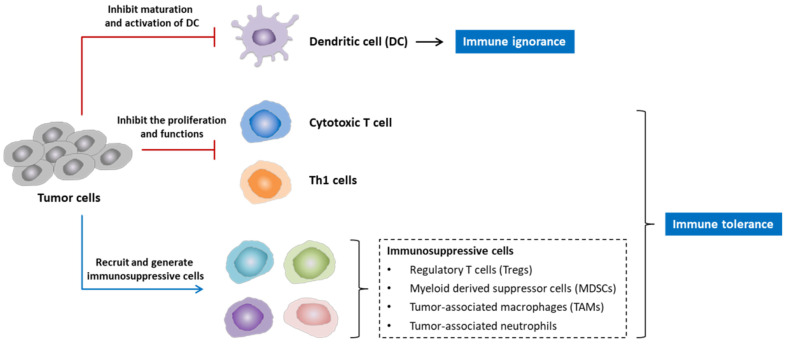
Immunosuppressive effects of tumor microenvironment (TME). Tumor cells secrete a number of chemokines and cytokines that inhibit immune cell population, including dendritic cell (DC) and T cells. The inhibition of DC for taking up and presenting tumor antigen may cause immune ignorance. Tumor cells also recruit and generate immunosuppressive cells, such as regulatory T cells (Tregs), myeloid-derived suppressor cells (MDSCs), tumor-associated macrophages (TAMs), and tumor-associated neutrophils, forming an immunosuppressive network that protects tumors from anti-tumor immune response, which leads to a condition called immune tolerance (modified from the presentation of Daniel W. Sharp et al. [69]).

The tumor microenvironment (TME) is the environment surrounding the tumor that consists of a variety of cell types, such as immune cells, the extracellular matrix, blood vessels, and cancer-associated fibroblasts. The interaction between TME and cancer cells profoundly influences various cellular processes, including tumor growth, invasion, and metastasis [63]. TME is frequently immunosuppressive, which can lead to two conditions—immune ignorance and immune tolerance. Therefore, TME plays an important role in the response of tumors to cancer immunotherapies. Several mechanisms are responsible for the immunosuppressive effects in TME. Chemokines and cytokines secreted by the tumors can inhibit dendritic cell (DC) maturation and activation that involve antigen-presenting cell (APC) recognition and presenting tumor antigens (immune ignorance). These molecules also cause a negative impact on the proliferation and function of cytotoxic T cells and Type 1 helper cells (Th1 cells). Moreover, tumor cells can promote the generation and activation of immunosuppressive cell populations, including regulatory T cells (Tregs), myeloid-derived suppressor cells (MDSCs), tumor-associated macrophages (TAMs), and tumor-associated neutrophils, leading to the downregulation of immune responses (immune tolerance). Subsequently, tumors are protected from anti-tumor immune response (Figure 3).

Tregs are a highly immune suppressive subset of CD4+ T cells expressing the transcription factor forkhead box P3 (FoxP3). Sakaguchi et al. identified Tregs as CD4+CD25+ T cells, and discovered specific expression of Foxp3 in Tregs. CD4+CD25+Foxp3+ is currently considered to be a classical combined marker of Treg cells. Foxp3 dominantly controls Tregs function and its suppressive capacity [64]. The depletion of Tregs or mutations in Foxp3 may lead to autoimmune disorders and allergy in vivo and humans. In the tumor microenvironment (TME), Tregs and other immune suppressive cytokines and molecules create an immunosuppressive environment to inhibit anti-tumor immunity, promoting the occurrence and development of tumors [65]. Therefore, Tregs are closely associated with the progression and prognosis of tumors [64].

Several suppressive mechanisms of Tregs have been identified, indicating the crucial roles of Tregs in the regulation of homeostasis of immune system and immune tolerance. Tregs inhibit immune function by secretion of inhibitory cytokines, such as IL-10, TGF-β, and IL-35, or through inhibition of CD8+ T cell and DC function through membrane-bound TGF-β. Tregs directly destroy effector cells that mediate cytotoxicity of CTLs, natural killer cells, and other cells through granzymes and perforin. In addition, Tregs also affect effector cell function by interfering with cell metabolism with different mechanisms. Treg cells modulate maturation and function of DCs via two main mechanisms. The interaction of cytotoxic T-lymphocyte antigen 4 (CTLA4) with CD80 and CD86 on the surface of DCs induces the release of indoleamine 2,3-dioxygenase (IDO), which is an immunosuppressive molecule, inhibiting T cell capacity. Additionally, the binding of lymphocyte-activation gene 3 (LAG3) to MHC class II molecules inhibits DC maturation and function [64,66]. VV has been reported to possess the ability to modulate TME and evade immunosuppression in TME via two major mechanisms [67]. Poxviruses including VV are able to escape from the host immune system by producing proteins that resemble cytokines, chemokines (virokines), and their receptors (viroceptors). These virokines and viroceptors can regulate different aspects of the host immune system and thus perturb normal host responses, which facilitates the prolonged virus infection and replication. Furthermore, the generation of these proteins may disrupt tumor-mediated cytokine signaling, which may help to evade the immunosuppression within TME, and subsequently allow immune cell recruitment to target tumor cells. Apart from the direct mechanism to modulate TME, VV can also indirectly cause cancer cell death. The infection of tumor cells by VV releases many pro-inflammatory signals and various TAAs that can lead to disruption of local vasculature and initiate innate and adaptive immune responses.

However, early OV-based therapy using wild-type VV has shown modest effects in cancer treatment. Numerous strategies have been developed to increase the effectiveness of VV-based therapeutic therapies and evade immunosuppressive effects of TME, suggesting that recombinant VVs that are generated by encoding immunostimulatory molecules and/or TAAs would be promising therapeutic options for cancer treatment [68,69,70]. As Tregs have caused a major obstacle for effective anti-tumor immunity, the combination of Tregs-targeting therapies and other modalities, such as ICIs or cancer vaccines, becomes promising to improve immunosuppressive TME [64]. Several studies have reported the positive effects of VV-based therapies on the inhibition and elimination of Treg cells. Novel fusogenic oncolytic vaccinia virus (FUVAC) was generated by Motomu Nakatake et al. during plaque purification of the mitogen-activated protein kinase-dependent recombinant vaccinia virus (MDRVV). FUVAC exhibited improved direct oncolytic activity and indirect anti-tumor immunity. FUVAC was reported to significantly inhibit tumor growth and improve the tumor immune microenvironment by reducing the tumor-associated immune suppressive cells, such as Treg cells, TAMs, and M-MDSCs, and increasing cytotoxic CD8+ T cells systemically [71]. In another study, VV was demonstrated to infect tumor infiltrating Tregs, leading to the depletion of viral-mediated Tregs, which is required for tumor regression [72].

### 3.3. Vaccinia Virus for Cancer Treatment

Oncolytic VV has been intensively investigated in many preclinical and clinical studies and has shown promising results [73,74]. The therapeutic efficacy of VV can be further enhanced via genetic engineering by replacing the vTk or VGF gene with targeted therapeutic genes or via the combination with different cancer therapies, such as chemotherapy or radiotherapy [19,61]. In our previous study, we designed a novel oncolytic virus (NOV) by substituting both the vTk and VGF regions with TRAIL and Ang1, respectively, in the VV Wyeth strain genome. Subsequently, the anti-tumor activity of NOV was dramatically enhanced, which was shown in the increased oncolytic efficacy in 14 cancer cell lines and in a colorectal cancer (CRC) syngeneic mouse model [19].

The oncolytic VVs, armed with T cell engagers, consisting of two single-chain variable fragments specific for CD3 and the tumor cell surface antigen EphA2, significantly improved anti-tumor activity of VV in lung cancer cell line A549 [74].

A recombinant VV, GLV-1h68, constructed by inserting three gene expression cassettes for a Renilla luciferase–Aequora green fluorescent fusion protein (RUC-GFP), β-galactosidase, and β-glucuronidase into the F14.5 L, J2R (encoding thymidine kinase), and A56R (encoding hemagglutinin) loci, respectively, was demonstrated to reduce toxicity and enhance tumor targeting specificity compared with its parental Lister strains [75]. In another study, GLV-1h68 was demonstrated to effectively infect, replicate in, and lyse human pancreatic tumor cell lines in vitro. Furthermore, a single intravenous dose of GLV-1h68 was able to effectively treat subcutaneous PANC-1 pancreatic tumor xenografts with minimal toxicity.

The therapeutic effects on PANC-1 tumor treatment were augmented and accelerated when combining GLV-1h68 with cisplatin or gemcitabine compared with the virus treatment alone. These findings indicated the outstanding therapeutic effects and safety profile of the recombinant VV GLV-1h68 to treat human pancreatic tumors in mice [76]. Combination with GLV-1h68 and the chemotherapy drug Cyclophosphamide significantly enhanced anti-tumor potency in human lung adenocarcinoma cell line PC14PE6 and exhibited synergistic anti-tumor effects on PC14PE6-RFP xenograft mouse models [61]. Additionally, the combination therapy of oncolytic VV strain GLV-1h68 and a β-galactosidase-activatable prodrug seco-analog of duocarmycin SA exhibited the induction of apoptosis in human breast cancer cell line GI-101A and showed beneficial effects on tumor regression in a breast cancer xenograft mouse model [77].

Another recombinant VV, hyper-IL-6-encoding VV strain GLV-1h90, was used in a combination therapy with the chemotherapeutic agent mitomycin C to treat DU-145 prostate xenograft tumors, resulting in significant improvement in the anti-tumor effects of the oncolytic virotherapy and reduction in chemotherapy-induced side effects, including thrombocytopenia [78].

The combination of oncolytic VV and radiotherapy was also investigated in several studies. It was reported that combining oncolytic VV strain GLV-1h68 with tumor-targeted ionizing radiation (IR) resulted in an increase in tumor regression and mouse survival in glioma tumor model [79]. Moreover, the combination treatment of a recombinant VV GLV-1h151 and radiation showed synergistic anti-tumor effects in vitro in human pancreatic cancer cell lines and significant inhibition of tumor growth in human pancreatic tumor xenograft mouse model without exhibiting any signs of toxicity. These data provided the evidence that the oncolytic VV can enhance the efficacy of radiotherapy and reduce treatment-associated toxicity [80].

These results showed the great potential of oncolytic VV in treating a wide range of human cancers, including pancreatic, prostate, lung, breast, colorectal cancer, or glioma. VV-based cancer therapy is, therefore, a promising strategy to bring more benefits to cancer treatment.

### 3.4. Vaccinia Virus for Cancer Vaccines in NSCLC

The therapeutic cancer vaccines for NSCLC have been investigated, and they provided positive results in murine tumor models and in phase I and II clinical trials, but achieved insignificant benefits in phase III clinical trials. There are several factors responsible for the failure of cancer vaccines for NSCLC, including the advanced stage of the disease, the choice of antigens or adjuvants, tumor-induced immunosuppression in the tumor microenvironment, or immunosenescence [15,17]. It is likely that the immune response induced by cancer vaccines is not strong enough to trigger therapeutic anti-tumor effects.

An ideal cancer vaccine should be able to elicit both potent CD4+ and CD8+ T cell responses and overcome tumor-induced immune tolerance. In order to achieve the expected outcome, the vaccine should consist of antigens that are specific to cancer cells (tumor-specific antigens—TSAs) or antigens that are expressed differently than in normal cells (tumor-associated antigens—TAAs). There have been discoveries of tumor antigens for the development of cancer vaccines, but many of them are poorly immunogenic, and thus lack clinical efficacy. Therefore, the presence of adjuvants in the formulation of cancer vaccines play an important role as they are able to induce strong and long-lasting immune responses or provide a delivery system for improved antigen presentation and activation of the immune cascade [13,16,23]. More importantly, since most of the patients with NSCLC are diagnosed at an old age and the immune system tends to decline with age, the safety and effectiveness of adjuvants should be considered carefully.

In a recent study, a virus-infected reprogrammed somatic cell-derived tumor cell vaccination (VIReST) regimen was suggested for the treatment and prevention of lung cancer development. Adenovirus or VV was used as an adjuvant which pre-infected the stem cell-derived lung cancer cells prior to the delivery of a prime–boost vaccination regimen to induce a potent anti-tumor immunity, which led to significantly prolonged survival in in a murine-inducible transgenic model of lung cancer and subcutaneous post-vaccine challenge models [15]. The concept of pre-infection using adenovirus and VV as a boost prior to vaccine delivery to improve vaccine immunogenicity was confirmed in a previous study [81]. The sequential treatment with adenovirus followed by VV was demonstrated to show more powerful anti-tumor efficacy when administered as therapeutics in animal tumor models compared to the use of virus alone [82]. Lemay CG et al. also demonstrated that pre-infection of tumor cells with an oncolytic virus, vesicular stomatitis virus (VSV), prior to the delivery of vaccine, provoked higher levels of anti-tumor immune response that was not achieved when vaccine was delivered to cells without pre-infection [83]. A recent article in Nature Communications reported that VV can be used as an adjuvant to boost antigen-specific immunity, and thus some OVs, including VV, can serve as adjuvant platforms in a prime–boost regimen for personalized anti-cancer vaccination. This research supports the possibility of using OVs-based therapies to accurately tailor to individuals with mutation-associated NSCLC [26].

### 3.5. Adverse Effects of Vaccinia Virus in Cancer Treatment

VV has been used for a long time; however, the adverse effects of VV as smallpox vaccine are rare, with about 0.1% including vaccinia necrosum, encephalitis, and eczema vaccinatum. In cancer treatment, the application of VV has been demonstrated to be well tolerated in a variety of studies. Significant vector-related toxicity was not observed when VV was delivered at different forms, including subcutaneous, intramuscular, intratumoral, and intravesical (bladder) injections. Mild vector-related toxicities were observed only at high doses [54]. In a phase I trial to determine the safety of GL-ONC1, a modified VV, when delivered as an intravenous infusion with chemoradiotherapy to patients with locally advanced head and neck cancer. In 19 enrolled patients, the most frequent adverse effects included grade 1–2 rigors, fever, fatigue, and rash; 6 patients experienced grade 3 adverse reactions, including hypotension, mucositis, nausea, and vomiting. No grade 4 acute toxicities were reported to be involved in GL-ONC1. Only 1 diabetic patient developed grade 4 hypoglycemia. The intravenous administration of GL-ONC1 was well tolerated in single and multiple escalating doses, and the delivery of GL-ONC1 is safe and feasible in selected patients [84]. Taken together, the application of VV is remarkably safe for cancer treatment.

### 3.6. Future Directions for Using Vaccinia Virus as Lung Cancer Vaccines

An analysis of the global clinical immuno-oncology (IO) landscape in 2017 showed that the number of cancer vaccines under clinical development is more than any other classes of IO, with more than 340 agents in clinical trials and another estimated 260 agents in preclinical and discovery stages [85]. The challenges of current cancer vaccines for NSCLC have created many opportunities for the development of cancer vaccines in the future. There will be a number of strategies to improve the clinical efficacy of cancer vaccines, such as the discovery and characterization of new tumor-specific antigens (neoantigens), adjuvants, delivery vectors, or administration methods. Among them, we focused the present view on the use of oncolytic VV in lung cancer vaccines.

“Oncolytic therapeutic vaccine” is a combination strategy utilizing OVs encoding one or more tumor-associated antigens or neoantigens with its oncolytic characteristics. The advantages of recombinant OVs have been explored in many preclinical and clinical studies. Vaccinia virus (VV) has been used as a novel platform for the attachment of tumor-specific peptides to induce a strong T cell-specific immune response toward these tumor antigens. The method is effective to enhance the treatment efficacy in melanoma mouse models [29]. Recombinant VVs created by engineering the VV to express TAAs and/or immune stimulatory molecules have been demonstrated to break immune tolerance, overcome immune inhibitory pathways, and induce strong anti-tumor immune responses. Since immunosuppressive TME is a big challenge to current immunotherapies in NSCLC patients, VV-based therapeutic vaccines are expected to improve anti-cancer therapeutic effects and clinical outcomes [17,69].

In a recent study, a new approach of cancer vaccination that used two different OVs encoding the same tumor antigen to prime and boost anti-tumor immunity was reported. Co-administering these viruses and peptide vaccines corresponding to cancer-specific mutations showed therapeutic efficacy in murine cancer models. OVs including VV were proposed for the use as adjuvant platforms for personalized cancer vaccines targeting specific mutations in patients [18]. A prime–boost strategy, using two different viral vectors, such as adenovirus and VV, has been proved to be highly effective in inducing anti-tumor immunity. This strategy not only maximizes tumor-specific immunity, but also circumvents antiviral immune responses. The host immune system against the viral vector may limit its potential to boost multiple times when using the same vector. The prime–boost vaccination regimen can be designed to include different OVs to boost anti-tumor immunity [54,69,82]. Given that NSCLC has high TMB, and many of these mutations have been identified, this strategy is a great choice to expand the treatment options for NSCLC.

## 4. Conclusions

Vaccinia virus possesses numerous advantages, equipping it to be an attractive and promising strategy for NSCLC treatment. With a long history of use as a smallpox vaccine and a viral vector or an adjuvant for treatment of various cancer types in many studies, the safety profile and therapeutic effects of VV have been firmly validated. Although positive results from early phases of clinical trials have been achieved in NSCLC patients, survival benefits in most phase III clinical trials has not yet been demonstrated. This challenge requires discoveries and identifications of new therapeutic strategies in cancer vaccine design that can surpass the hurdles to contribute to beneficial outcomes to NSCLC patients. The promising data from recent studies in NSCLC has brought a new option to NSCLC treatment, suggesting that VV is used as a therapeutic vaccine or a potent adjuvant platform for personalized cancer vaccination, which will create a new paradigm of lung cancer treatment and provide significant benefits in clinical setting.
